# The electronic and magnetic properties of functionalized silicene: a first-principles study

**DOI:** 10.1186/1556-276X-7-422

**Published:** 2012-07-28

**Authors:** Fu-bao Zheng, Chang-wen Zhang

**Affiliations:** 1 School of Physics and Technology, University of Jinan, Jinan, Shandong, 250022, People’s Republic of China

**Keywords:** First-principles calculation, Silicene, Ferromagnetism, Curie temperature

## Abstract

Based on first-principles calculations, we study the structural, electronic, and magnetic properties of two-dimensional silicene saturated with hydrogen and bromine atoms. It is found that the fully saturated silicene exhibits nonmagnetic semiconducting behavior, while half-saturation on only one side with hydrogen or bromine results in the localized and unpaired electrons of the unsaturated Si atoms, showing ferromagnetic semiconducting or half-metallic properties, respectively. Total energy calculations show that the half-hydrogenated silicene exhibits a ferromagnetic order, while the half-brominated one exhibits an antiferromagnetic behavior.

## Background

Recently, low-dimensional honeycomb graphene has attracted much interest because of its unique electronic properties as well as its potential applications in future nanoelectronics, and therefore is one of the most investigated materials in physics and nanoscience [1]. Nevertheless, graphene is facing many challenges in its growth over large areas and, importantly, incompatibility with current silicon-based electronic technology. As the counterpart of graphene, the two-dimensional (2D) hexagonal silicene [2] recently is chemically exfoliated from calcium disilicide (CaSi_2_). In the more recent works, Si nanoribbons are fabricated by deposition on a silver substrate [3,4]. The synthesis of silicon-based nanomaterials opens the way for studying their physical and chemical properties, with the added advantage of being compatible with existing semiconductor devices.

The chemical functionalization is generally an efficient way to tune the electronic and magnetic properties in 2D structures, such as graphene, BN, AlN, and CdS sheets [5-8]. Especially, on-plane chemical modification with hydrogen has been reported to induce long-range ferromagnetic order without 3*d* or 4*f* element doping in such 2D carbon-based materials [9,10], not suffering from problems related to precipitates or secondary phase formation in 3*d-* or 4*f*-element-doped materials, which are undesirable for practical applications. For Si-based nanostructures, Jose and Datta [11] reported the structures and electronic properties of silicene clusters and Si-substituted benzenes, suggesting that silicene clusters may be a promising material for FET and hydrogen storage. Since silicene has only recently been realized [4,5], the effects of adsorption of foreign atoms on the surface of silicene on magnetism have not been thoroughly explored. In the present letter, based on first-principles calculations, we focus on the possibility of realizing ferromagnetism in silicene with adsorption of hydrogen and the halogen element bromine (Br). It can be seen that the electronic properties of silicene can be tuned, and especially, the ferromagnetic order or half-metallicity is achieved upon adsorption of H and Br atoms, which may open a new route to design the silicon-based nanostructures in spintronics.

## Methods

All the predictions have been performed using the Vienna *Ab initio* Simulation Package and density functional theory [12]. The generalized gradient approximation [13] and a 450-eV cutoff energy for the plane-wave basis set were used. Pseudopotentials with 3*s*^2^3*p*^2^, 1 *s*^1^, and 4*s*^2^4*p*^5^ valence electron configurations for Si, H, and Br atoms were used, respectively. Following the Monkhorst-Pack scheme [14], Brillouin-zone integration was carried out at 9 × 9 × 1 k-points, and 15 × 15 × 1 k-points were used to obtain the electronic properties. The symmetry-unrestricted optimizations for geometry were performed using the conjugate gradient scheme until the largest Hellmann-Feynman force is smaller than 0.01 eV/Å.

## Results and discussion

**Figure 1:**
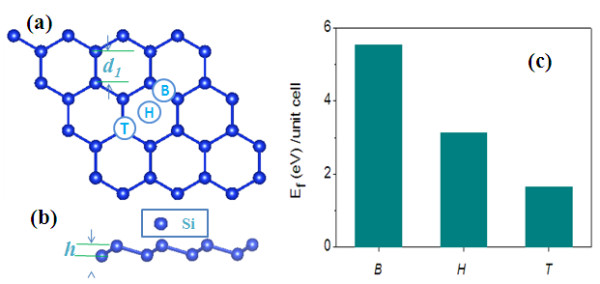
**Relaxed geometric structure of silicene and formation energies for different doping sites.** Relaxed geometric structure of silicene from the top view (**a**) and the side view (**b**). The formation energies for different doping sites are also given in (**c**).

The investigated model of silicene sheet is shown in Figure 1a. There are eight silicon atoms in the primitive cell. The calculated bond length of the Si-Si bond is *d*_1_ = 2.27 Å, which agrees well with the previous study [15]. Different from graphene, the larger Si-Si bond length weakens the π-π overlaps, resulting in a low-buckled structure (*h* = 0.45 Å) with *sp*^3^-like hybrid orbitals (Figure 1b). To check which site a single H atom can absorb on, we consider three different adsorption configurations on the silicene, i.e., top site (T), bridge site (B), and hollow site (H), as shown in Figure 1a. The relative stabilities of the structure are determined from the formation energy which are defined as *E*_f_ = *E*(H:silicene) – *E*(silicene) – 1/2*nμ*_H_, where *E*(H:silicene) and *E*(silicene) are the total energies of the supercell with and without the impurities, respectively. *μ*_H_ is the chemical potential of H_2_ gases, and *n* is the concentrations of H atoms in silicene. From our calculations, the formation energy for T is found to be the lowest, as shown in Figure 1c. Thus, the T site is the stable adsorption positions for the H atom, suggesting that the growth of hydrogenated silicene can make full use of deposition techniques, which enable the control of a 2D material to avoid the formation of 3D islands.

**Figure 2:**
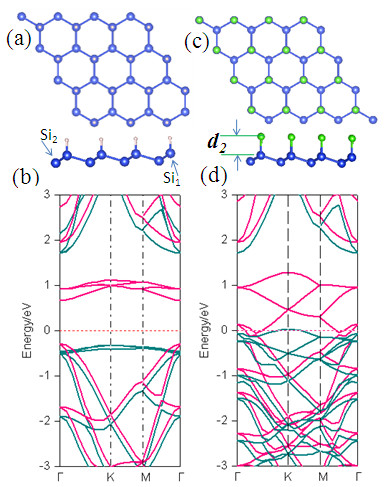
**Relaxed geometry and band structure for half-saturated H@Si**_**1**_**(a, b) and Br@Si**_**1**_**(c, d), respectively.** In the band structures, the green and pink lines correspond to spin-up and spin-down channels, respectively, and the energies are relative to the Fermi level and indicated by a red line.

When silicene lies on a substrate, it generally leads to a half-decorated sheet with only one side being functionalized by hydrogen, which can also be achieved by applying an external electric field perpendicularly to the (0001) surface in silicene. Thus, we consider the structure of half-hydrogenated silicene (H@Si_1_), where the top of Si_1_ atoms are hydrogenated and Si_2_ atoms remain unsaturated (Figure 2a) [16]. In the case of pure silicene, the *p*_*z*_ orbitals perpendicular to the plane of the Si ring system hybridize to form a weak and extensive π-bonding network. When half of the silicon (Si_1_) atoms are hydrogenated, the H atoms would form strong σ bonds with Si_1_ atoms, resulting in *sp*^3^ hybridization between hydrogen and Si atoms, while the Si_2_ atoms remain *sp*^2^ hybridized. These make the electrons in the unsaturated Si_2_ atoms localized and unpaired, leading to Si_2_ being spin-polarized with an integer magnetic moment per unit cell. To check whether the magnetic order is collective, the energy difference, between ferromagnetic and antiferromagnetic, is found to be 0.068 eV, and thus the ferromagnetic order is the stable ground state. We predicted the Curie temperature with the formula of $\gamma k_{\text{B}}{\text{T}}_{\text{C}}/2\;=E_{\text{AFM}}–E_{\text{FM}}$ from mean-field approximation [17], where *γ* is the structural dimension, and *k*_B_ is the Boltzmann constant. We found that the calculated Curie temperature of the configuration H@Si_1_ is about 300 K, which is ideal in practical applications in spintronics.

**Figure 3:**
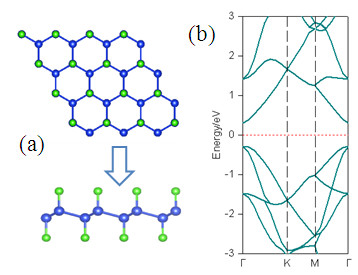
The calculated crystal structure (a) and the corresponding band structure for fully brominated silicene (b).

Recent studies show that surface saturation with halogen elements is an effective way to modulate the electronic properties of Si nanowires [18]. Zhou et al. [19] also show that fluorine atoms decorated on graphene or carbon nanotube on different sites can induce desirable magnetic properties. More recently, Yaya et al. [20] study the bromination in graphene and graphite, and predict the intriguing electronic properties. Therefore, it is very interesting to investigate whether the ferromagnetic properties can be induced by the adsorption of the halogen element Br, as an example. When silicene is fully brominated on both sides (Figure 3a), we find that it exhibits nonmagnetic semiconducting behaviors with a direct bandgap of 1.47 eV (Figure 3b), smaller than that of hydrogenated silicene [21].

In the case of half-brominated silicene (Br@Si_1_), Bader analysis shows that it is spin-polarized with a local magnetic moment of 1.0 *μ*_B_ per unit cell, similar with that of H@Si_1_. More interestingly, the energy bands close to the Fermi level show a metallic spin-down channel and a semiconducting spin-up one with a 1.73-eV bandgap, and thus a half-metallic behavior with 100% spin-polarized current is obtained, suggesting a feasible way of building spin devices based on silicene. To determine the magnetic stability of Br-induced half-metallicity in Br@Si_1_, the total energy differences of ferromagnetic, antiferromagnetic, and nonmagnetic orders are calculated. We find that the antiferromagnetic state lies 0.17 and 0.51 eV lower per unit cell in energy than ferromagnetic and nonmagnetic states, respectively, indicating that Br@Si_1_exhibits an antiferromagnetic behavior.

**Figure 4:**
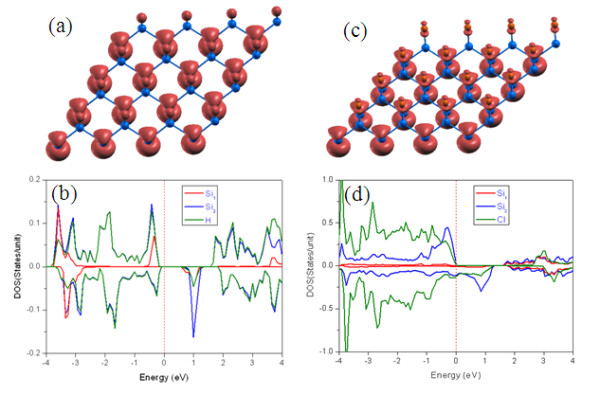
**Spatial spin-density distribution and PDOS for half-saturated H@Si**_**1**_**(a, b) and Br@Si**_**1**_**(c, d), respectively.**

To illustrate the origin behind magnetic properties in the *M*@Si_1_ (*M* = H or Br) sheets, the project density of states (PDOS) and isosurface of spin density are presented in Figure 4. The local magnetic moments are mainly contributed by the 3*p* electrons near the Fermi level of unsaturated Si_2_ atoms, i.e., 0.27 and 0.24 *μ*_B_ for H@Si_1_ and Br@Si_1_, respectively, while the saturated Si_1_ atom carries a very small magnetic moment (Figure 4). However, the adsorbed Br atom in Br@Si_1_ provides a magnetic moment of 0.11 *μ*_B_, larger than that (0.05 *μ*_B_) of the H atom in H@Si_1_. Recently, John et al. [22] investigated the magnetic interactions in layered nickel alkanethiolates and a dinuclear Ni(II) complex. They found that the overall magnetic behavior of the system depends on the delicate balance between the competing ferromagnetic and antiferromagnetic interactions. However, in H@Si_1_, since the valence electrons in 3*p*-states on Si_2_ are more delocalized than those in *d*- or *f*-states, the larger spatial extension promotes long-range exchange ferromagnetic coupling, due to the extended *p-p* interactions. In fact, the extended tails of wave functions have also been proposed to mediate long-range ferromagnetism in nonmagnetic element-doped nanostructures 5.

## Conclusions

In summary, based on first-principles calculations, we study the electronic structure and magnetic properties of 2D hexagonal silicene adsorbed with H and Br atoms. We find that the fully saturated silicene on both sides exhibits nonmagnetic semiconducting behaviors. For half-saturation on only one side of silicene, H@Si_1_ exhibits a ferromagnetic behavior, while Br@Si_1_ shows a half-metallic property due to the localized and unpaired electrons of unsaturated Si_2_ atoms. Calculations of total energies show that Br@Si_1_ exhibits an antiferromagnetic behavior, while H@Si_1_ shows a long-range ferromagnetic order with a Curie temperature at about room temperature. Once combined with advanced Si nanotechnology, these predicted properties may be very useful as a promising nanoscale technological application in spintronics. Therefore, our work suggests that it may be possible to realize long-range room-temperature ferromagnetism in silicene sheets and may motivate potential applications of Si-based nanostructures in spintronics.

## Competing interests

The authors declare that they have no competing interests.

## Authors’ contributions

CWZ conceived the idea and designed the calculated model. FBZ carried out the electronic structure calculations and data analysis. Both authors read and approved the final manuscript.

## Authors’ information

FBZ is a graduate student and CWZ is a professor in the School of Physics and Technology, University of Jinan, Shandong, People's Republic of China.

## References

[B1] Geim AK Novoselov KS The rise of graphene Nature Mater 2007 6 183 191 10.1038/nmat1849 17330084

[B2] Nakano H Mitsouka T Harada M Horibuchi K Nozaki H Takahashi N Nonaka T Seno Y Nakamura H Soft synthesis of single-crystal silicon monolayer sheets Angew Chem 2006 118 6451 6454 10.1002/ange.200600321 16941712

[B3] Aufray B Kara A Vizzini S Oughaddou H Léandri C Ealet B Lay GL Graphene-like silicon nanoribbons on Ag (110): a possible formation of silicene Appl Phys Lett 2010 96 183102 10.1063/1.3419932

[B4] De Padova P Quaresima C Ottaviani C Sheverdyaeva PM Moras P Carbone C Topwal D Olivieri B Kara A Oughaddou H Aufray B Lay GL Evidence of graphene-like electronic signature nanoribbons Appl Phys Lett 2010 96 261905 10.1063/1.3459143

[B5] Zhang CW Yan SS Wang PJ Li P Zheng FB First-principles study on the electronic and magnetic properties of hydrogenated CdS nanosheets J Appl Phys 2011 109 094304 10.1063/1.3583659

[B6] Golberg D Bando Y Huang Y Terao T Mitome M Tang CC Zhi CY Boron nitride nanotubes and nanosheets ACS Nano 2010 4 6 2979 2993 10.1021/nn1006495 20462272

[B7] Zhou J Wang Q Sun Q Jena P Electronic and magnetic properties of a BN sheet decorated with hydrogen and fluorine Phys Rev B 2010 81 085442

[B8] Zhang CW Zheng FB First-principles prediction on electronic and magnetic properties of hydrogenated AlN nanosheets J Comput Chem 2011 32 3122 3128 10.1002/jcc.21902 21815179

[B9] Wang Y Ding Y Shi S Tang W Electronic structures of graphane sheets with foreign atom substitutions Appl Phys Lett 2011 98 163104 10.1063/1.3574906

[B10] Lu N Li ZY Yang JL Electronic structure engineering via on-plane chemical functionalization: a comparison study on two-dimensional polysilane and graphane J Phys Chem C 2011 113 16741 16746

[B11] Jose D Datta A Molecular rotor inside a phosphonate cavitand: role of supramolecular interactions Phys Chem Chem Phys 2010 13 7237

[B12] Kresse G Hafner J Ab initio molecular dynamics for liquid metals Phys Rev B 1993 47 558 561 10.1103/PhysRevB.47.558 10004490

[B13] Kresse G Joubert D From ultrasoft pseudopotentials to the projector augmented-wave method Phys Rev B 1999 59 1758 1775 10.1103/PhysRevB.59.1758

[B14] Monkhorst HJ Pack JD Special points for Brillouin-zone integrations Phys Rev B 1976 13 5188 5192 10.1103/PhysRevB.13.5188

[B15] Lebègue S Eriksson O Electronic structure of two-dimensional crystals from ab initio theory Phys Rev B 2009 79 115409(1) 115409(4)

[B16] Cahangirov S Topsakal M Akturk E Sahin H Ciraci S Two- and one-dimensional honeycomb structures of silicon and germanium Phys Rev Lett 2009 102 236804(1) 236804(4) 19658958 10.1103/PhysRevLett.102.236804

[B17] Kudrnovsky J Turek I Drchal V Maca F Weinberger P Bruno P Exchange interactions in III-V and group-IV diluted magnetic semiconductors Phys Rev B 2004 69 115208(1) 115208(11)

[B18] Zhou J Wang Q Sun Q Chen XS Kawazoe Y Jena P Ferromagnetism in semihydrogenated graphene sheet Nano Lett 2009 9 3867 3870 10.1021/nl9020733 19719081

[B19] Zhou J Wu M Zhou X Sun Q Tuning electronic and magnetic properties of graphene by surface modification Appl Phys Lett 2009 95 103108 10.1063/1.3225154

[B20] Yaya A Ewels CP Suarez-Martinez I Wagner P Lefrant S Okotrub A Bulusheva L Briddon PR Bromination of graphene and graphite Phys Rev B 2011 83 045411

[B21] Gao N Zheng WT Jiang Q Density functional theory calculations for two-dimensional silicene with halogen functionalization Phys Chem Chem Phys 2012 14 257 261 22083171 10.1039/c1cp22719j

[B22] John NS Kulkarni GU Datta A Pati SK Komori F Kavitha G Narayana C Sanyal MK Magnetic interactions in layered nickel alkanethiolates J Phys Chem C 2007 111 1868 1870 10.1021/jp0675072

